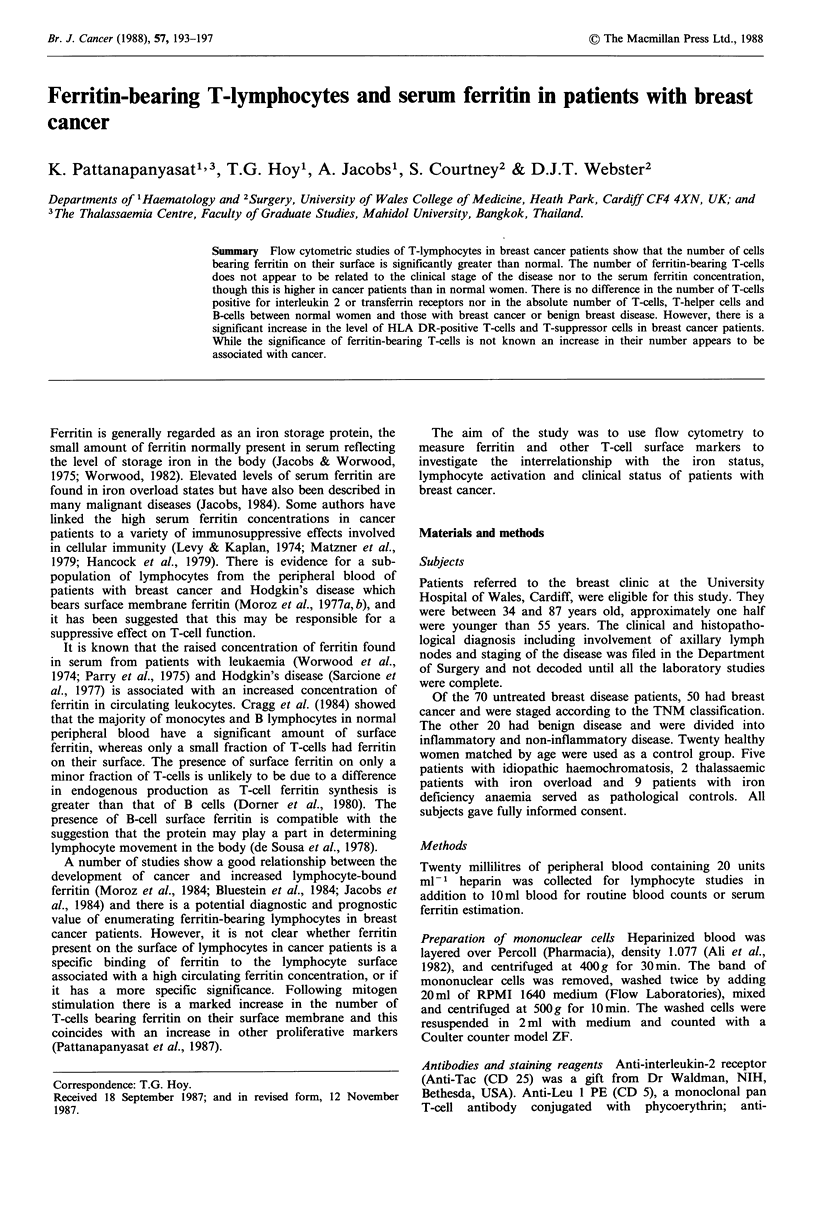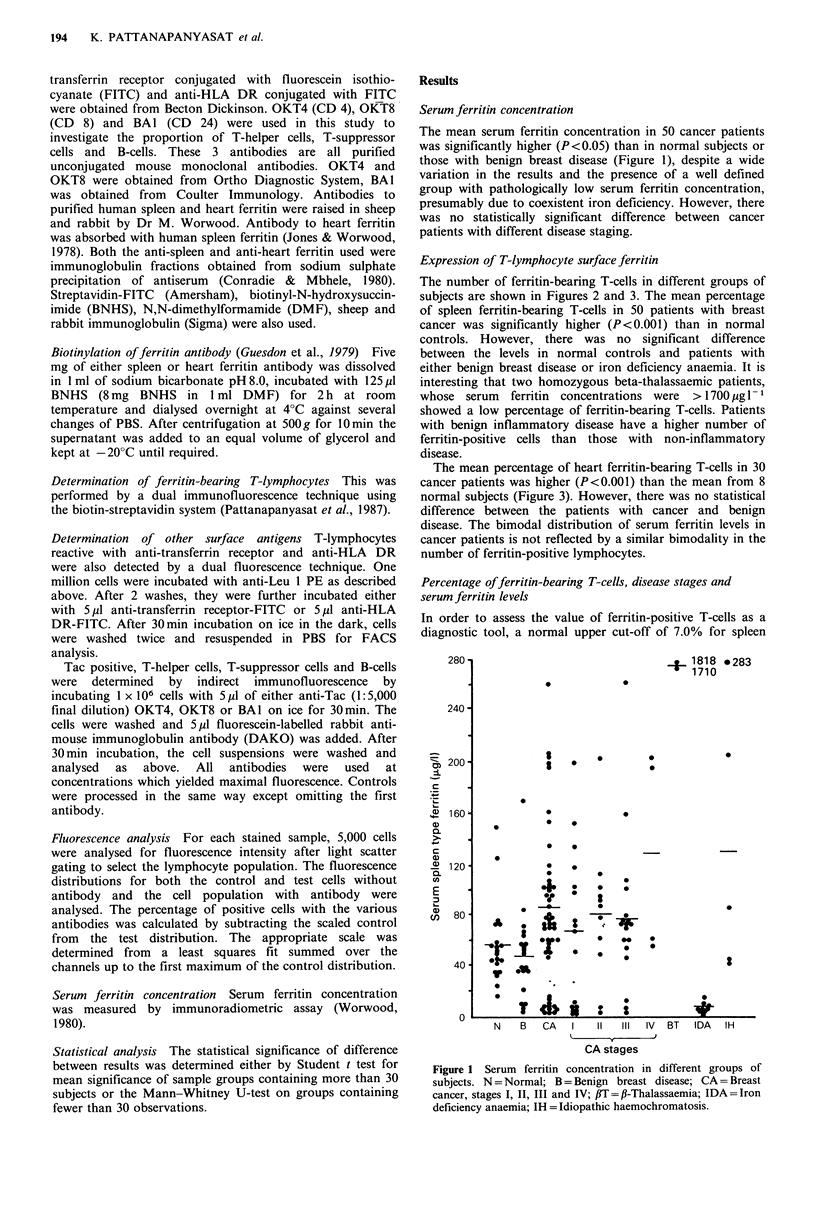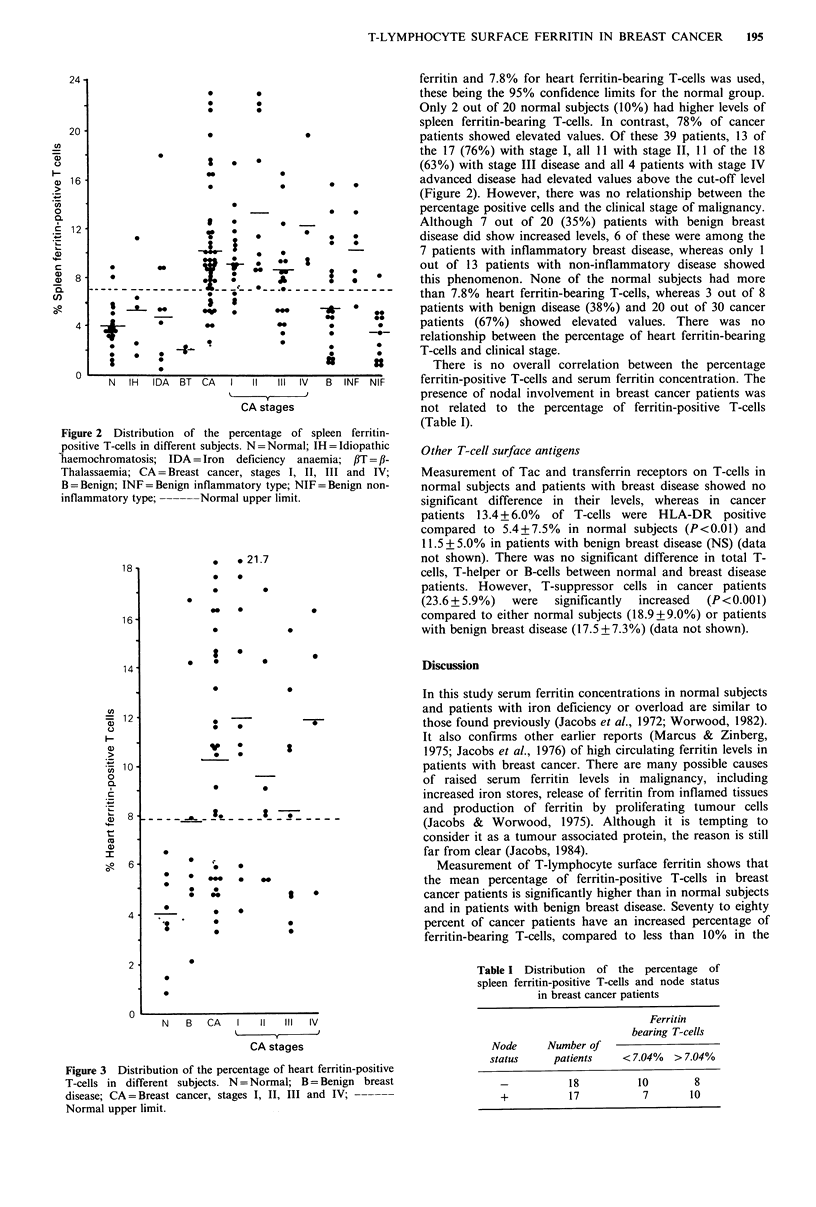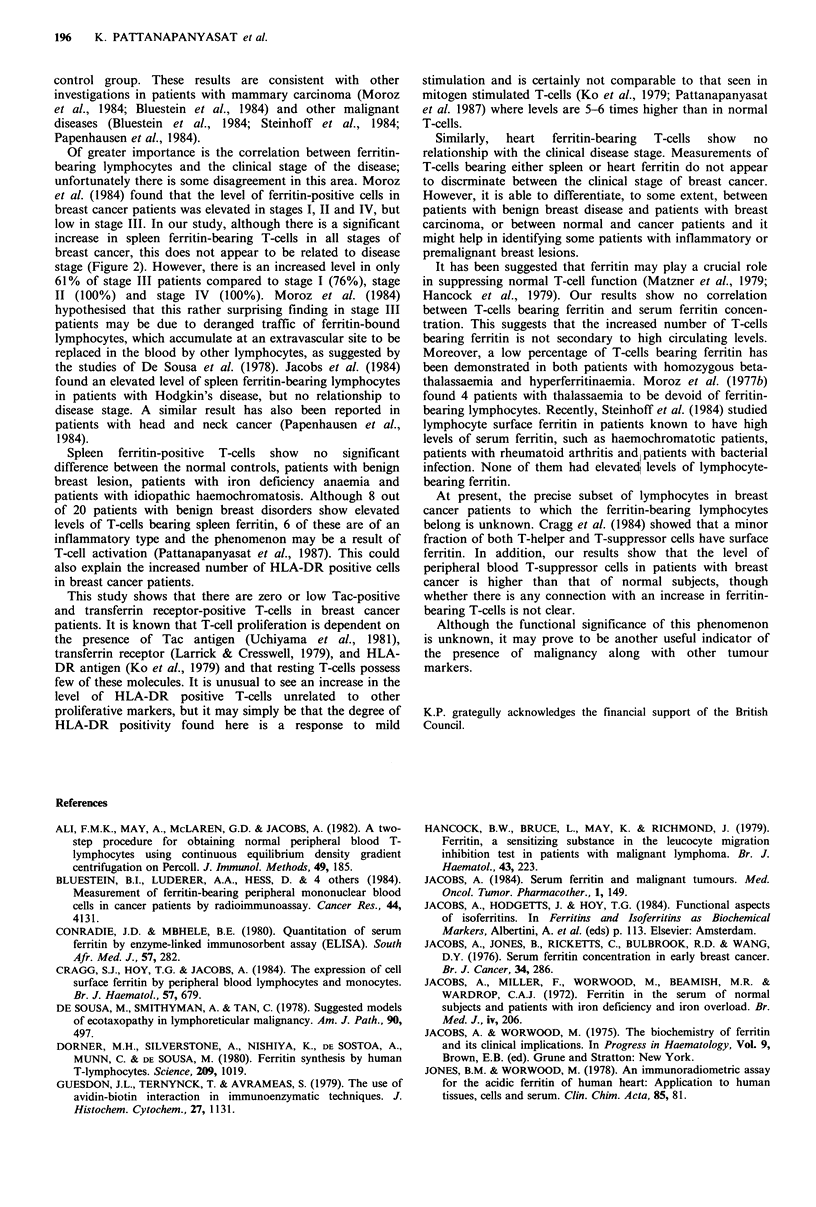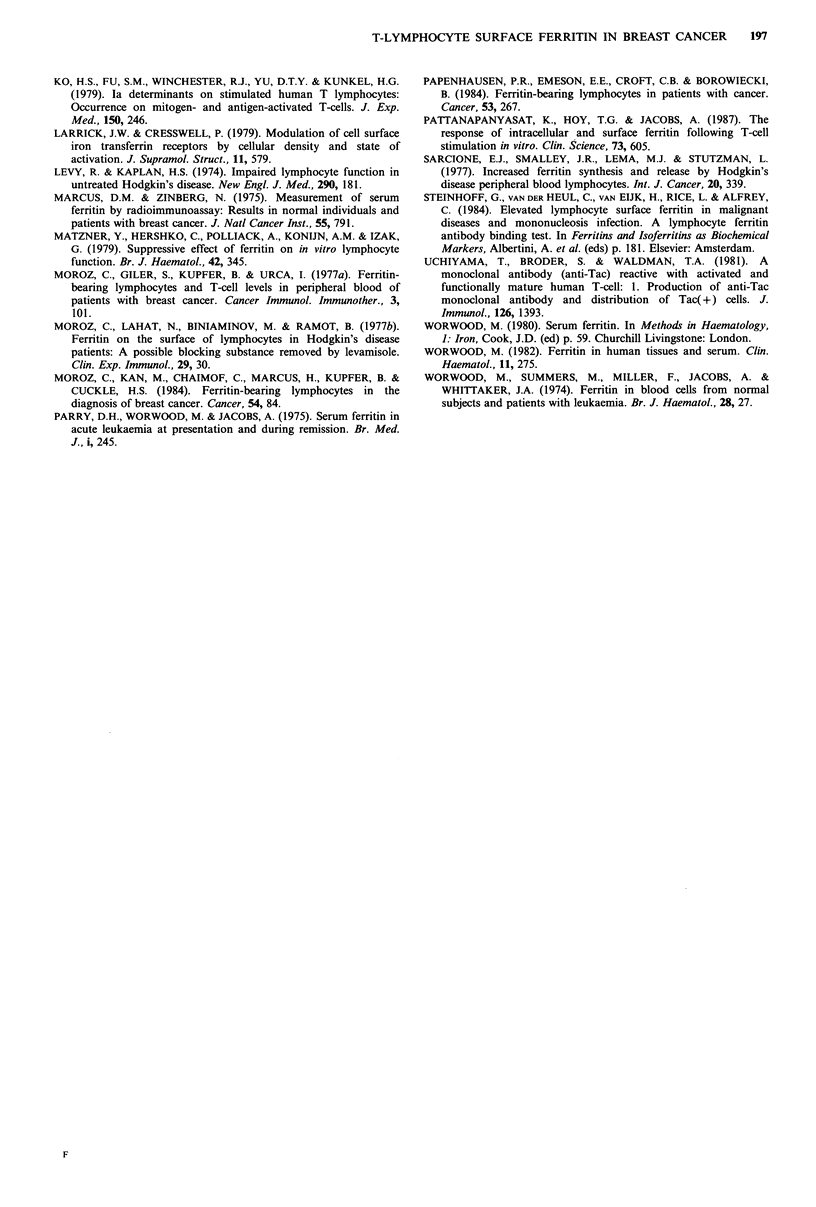# Ferritin-bearing T-lymphocytes and serum ferritin in patients with breast cancer.

**DOI:** 10.1038/bjc.1988.41

**Published:** 1988-02

**Authors:** K. Pattanapanyasat, T. G. Hoy, A. Jacobs, S. Courtney, D. J. Webster

**Affiliations:** Department of Haematology, University of Wales College of Medicine, Health Park, Cardiff, UK.

## Abstract

Flow cytometric studies of T-lymphocytes in breast cancer patients show that the number of cells bearing ferritin on their surface is significantly greater than normal. The number of ferritin-bearing T-cells does not appear to be related to the clinical stage of the disease nor to the serum ferritin concentration, though this is higher in cancer patients than in normal women. There is no difference in the number of T-cells positive for interleukin 2 or transferrin receptors nor in the absolute number of T-cells, T-helper cells and B-cells between normal women and those with breast cancer or benign breast disease. However, there is a significant increase in the level of HLA DR-positive T-cells and T-suppressor cells in breast cancer patients. While the significance of ferritin-bearing T-cells is not known an increase in their number appears to be associated with cancer.


Br. J. Cancer (1988), 57, 193–197 © The Macmillan Press Ltd., 1988

### Ferritin-bearing T-lymphocytes and serum ferritin in patients with breast cancer

K. Pattanapanyasat[1,3], T.G. Hoy[1], A. Jacobs[1], S. Courtney[2] & D.J.T. Webster[2]

*Departments of [1]Haematology and [2]Surgery, University of Wales College of Medicine, Heath Park, Cardiff CF4 4XN, UK; and [3]The Thalassaemia Centre, Faculty of Graduate Studies, Mahidol University, Bangkok, Thailand.*

**Summary** Flow cytometric studies of T-lymphocytes in breast cancer patients show that the number of cells bearing ferritin on their surface is significantly greater than normal. The number of ferritin-bearing T-cells does not appear to be related to the clinical stage of the disease nor to the serum ferritin concentration, though this is higher in cancer patients than in normal women. There is no difference in the number of T-cells positive for interleukin 2 or transferrin receptors nor in the absolute number of T-cells, T-helper cells and B-cells between normal women and those with breast cancer or benign breast disease. However, there is a significant increase in the level of HLA DR-positive T-cells and T-suppressor cells in breast cancer patients. While the significance of ferritin-bearing T-cells is not known an increase in their number appears to be associated with cancer.

Ferritin is generally regarded as an iron storage protein, the small amount of ferritin normally present in serum reflecting the level of storage iron in the body (Jacobs & Worwood, 1975; Worwood, 1982). Elevated levels of serum ferritin are found in iron overload states but have also been described in many malignant diseases (Jacobs, 1984). Some authors have linked the high serum ferritin concentrations in cancer patients to a variety of immunosuppressive effects involved in cellular immunity (Levy & Kaplan, 1974; Matzner *et al.*, 1979; Hancock *et al.*, 1979). There is evidence for a subpopulation of lymphocytes from the peripheral blood of patients with breast cancer and Hodgkin's disease which bears surface membrane ferritin (Moroz *et al.*, 1977*a, b*), and it has been suggested that this may be responsible for a suppressive effect on T-cell function.

It is known that the raised concentration of ferritin found in serum from patients with leukaemia (Worwood *et al.*, 1974; Parry *et al.*, 1975) and Hodgkin's disease (Sarcione *et al.*, 1977) is associated with an increased concentration of ferritin in circulating leukocytes. Cragg *et al.* (1984) showed that the majority of monocytes and B lymphocytes in normal peripheral blood have a significant amount of surface ferritin, whereas only a small fraction of T-cells had ferritin on their surface. The presence of surface ferritin on only a minor fraction of T-cells is unlikely to be due to a difference in endogenous production as T-cell ferritin synthesis is greater than that of B cells (Dorner *et al.*, 1980). The presence of B-cell surface ferritin is compatible with the suggestion that the protein may play a part in determining lymphocyte movement in the body (de Sousa *et al.*, 1978).

A number of studies show a good relationship between the development of cancer and increased lymphocyte-bound ferritin (Moroz *et al.*, 1984; Bluestein *et al.*, 1984; Jacobs *et al.*, 1984) and there is a potential diagnostic and prognostic value of enumerating ferritin-bearing lymphocytes in breast cancer patients. However, it is not clear whether ferritin present on the surface of lymphocytes in cancer patients is a specific binding of ferritin to the lymphocyte surface associated with a high circulating ferritin concentration, or if it has a more specific significance. Following mitogen stimulation there is a marked increase in the number of T-cells bearing ferritin on their surface membrane and this coincides with an increase in other proliferative markers (Pattanapanyasat *et al.*, 1987).

Correspondence: T.G. Hoy.
Received 18 September 1987; and in revised form, 12 November 1987.

The aim of the study was to use flow cytometry to measure ferritin and other T-cell surface markers to investigate the interrelationship with the iron status, lymphocyte activation and clinical status of patients with breast cancer.

### Materials and methods

### *Subjects*

Patients referred to the breast clinic at the University Hospital of Wales, Cardiff, were eligible for this study. They were between 34 and 87 years old, approximately one half were younger than 55 years. The clinical and histopathological diagnosis including involvement of axillary lymph nodes and staging of the disease was filed in the Department of Surgery and not decoded until all the laboratory studies were complete.

Of the 70 untreated breast disease patients, 50 had breast cancer and were staged according to the TNM classification. The other 20 had benign disease and were divided into inflammatory and non-inflammatory disease. Twenty healthy women matched by age were used as a control group. Five patients with idiopathic haemochromatosis, 2 thalassaemic patients with iron overload and 9 patients with iron deficiency anaemia served as pathological controls. All subjects gave fully informed consent.

### *Methods*

Twenty millilitres of peripheral blood containing 20 units $ml^{-1}$ heparin was collected for lymphocyte studies in addition to 10 ml blood for routine blood counts or serum ferritin estimation.

*Preparation of mononuclear cells* Heparinized blood was layered over Percoll (Pharmacia), density 1.077 (Ali *et al.*, 1982), and centrifuged at 400 *g* for 30 min. The band of mononuclear cells was removed, washed twice by adding 20 ml of RPMI 1640 medium (Flow Laboratories), mixed and centrifuged at 500 *g* for 10 min. The washed cells were resuspended in 2 ml with medium and counted with a Coulter counter model ZF.

*Antibodies and staining reagents* Anti-interleukin-2 receptor (Anti-Tac (CD 25) was a gift from Dr Waldman, NIH, Bethesda, USA). Anti-Leu 1 PE (CD 5), a monoclonal pan T-cell antibody conjugated with phycoerythrin; antitransferrin receptor conjugated with fluorescein isothiocyanate (FITC) and anti-HLA DR conjugated with FITC were obtained from Becton Dickinson. OKT4 (CD 4), OKT8 (CD 8) and BA1 (CD 24) were used in this study to investigate the proportion of T-helper cells, T-suppressor cells and B-cells. These 3 antibodies are all purified unconjugated mouse monoclonal antibodies. OKT4 and OKT8 were obtained from Ortho Diagnostic System, BA1 was obtained from Coulter Immunology. Antibodies to purified human spleen and heart ferritin were raised in sheep and rabbit by Dr M. Worwood. Antibody to heart ferritin was absorbed with human spleen ferritin (Jones & Worwood, 1978). Both the anti-spleen and anti-heart ferritin used were immunoglobulin fractions obtained from sodium sulphate precipitation of antiserum (Conradie & Mbhele, 1980). Streptavidin-FITC (Amersham), biotinyl-N-hydroxysuccinimide (BNHS), N,N-dimethylformamide (DMF), sheep and rabbit immunoglobulin (Sigma) were also used.

*Biotinylation of ferritin antibody* (*Guesdon* et al., *1979*) Five mg of either spleen or heart ferritin antibody was dissolved in 1 ml of sodium bicarbonate pH 8.0, incubated with 125 $\mu$l BNHS (8 mg BNHS in 1 ml DMF) for 2 h at room temperature and dialysed overnight at 4°C against several changes of PBS. After centrifugation at 500 *g* for 10 min the supernatant was added to an equal volume of glycerol and kept at −20°C until required.

*Determination of ferritin-bearing T-lymphocytes* This was performed by a dual immunofluorescence technique using the biotin-streptavidin system (Pattanapanyasat *et al.*, 1987).

*Determination of other surface antigens* T-lymphocytes reactive with anti-transferrin receptor and anti-HLA DR were also detected by a dual fluorescence technique. One million cells were incubated with anti-Leu 1 PE as described above. After 2 washes, they were further incubated either with 5 $\mu$l anti-transferrin receptor-FITC or 5 $\mu$l anti-HLA DR-FITC. After 30 min incubation on ice in the dark, cells were washed twice and resuspended in PBS for FACS analysis.

Tac positive, T-helper cells, T-suppressor cells and B-cells were determined by indirect immunofluorescence by incubating $1 \times 10^6$ cells with 5 $\mu$l of either anti-Tac (1:5,000 final dilution) OKT4, OKT8 or BA1 on ice for 30 min. The cells were washed and 5 $\mu$l fluorescein-labelled rabbit anti-mouse immunoglobulin antibody (DAKO) was added. After 30 min incubation, the cell suspensions were washed and analysed as above. All antibodies were used at concentrations which yielded maximal fluorescence. Controls were processed in the same way except omitting the first antibody.

*Fluorescence analysis* For each stained sample, 5,000 cells were analysed for fluorescence intensity after light scatter gating to select the lymphocyte population. The fluorescence distributions for both the control and test cells without antibody and the cell population with antibody were analysed. The percentage of positive cells with the various antibodies was calculated by subtracting the scaled control from the test distribution. The appropriate scale was determined from a least squares fit summed over the channels up to the first maximum of the control distribution.

*Serum ferritin concentration* Serum ferritin concentration was measured by immunoradiometric assay (Worwood, 1980).

*Statistical analysis* The statistical significance of difference between results was determined either by Student *t* test for mean significance of sample groups containing more than 30 subjects or the Mann–Whitney U-test on groups containing fewer than 30 observations.

### Results

*Serum ferritin concentration*

The mean serum ferritin concentration in 50 cancer patients was significantly higher ($P<0.05$) than in normal subjects or those with benign breast disease (Figure 1), despite a wide variation in the results and the presence of a well defined group with pathologically low serum ferritin concentration, presumably due to coexistent iron deficiency. However, there was no statistically significant difference between cancer patients with different disease staging.

*Expression of T-lymphocyte surface ferritin*

The number of ferritin-bearing T-cells in different groups of subjects are shown in Figures 2 and 3. The mean percentage of spleen ferritin-bearing T-cells in 50 patients with breast cancer was significantly higher ($P<0.001$) than in normal controls. However, there was no significant difference between the levels in normal controls and patients with either benign breast disease or iron deficiency anaemia. It is interesting that two homozygous beta-thalassaemic patients, whose serum ferritin concentrations were $>1700\,\mu g\,l^{-1}$ showed a low percentage of ferritin-bearing T-cells. Patients with benign inflammatory disease have a higher number of ferritin-positive cells than those with non-inflammatory disease.

The mean percentage of heart ferritin-bearing T-cells in 30 cancer patients was higher ($P<0.001$) than the mean from 8 normal subjects (Figure 3). However, there was no statistical difference between the patients with cancer and benign disease. The bimodal distribution of serum ferritin levels in cancer patients is not reflected by a similar bimodality in the number of ferritin-positive lymphocytes.

*Percentage of ferritin-bearing T-cells, disease stages and serum ferritin levels*

In order to assess the value of ferritin-positive T-cells as a diagnostic tool, a normal upper cut-off of 7.0% for spleen

**Figure 1** Serum ferritin concentration in different groups of subjects. N = Normal; B = Benign breast disease; CA = Breast cancer, stages I, II, III and IV; $\beta$T = $\beta$-Thalassaemia; IDA = Iron deficiency anaemia; IH = Idiopathic haemochromatosis.

**Figure 2** Distribution of the percentage of spleen ferritin-positive T-cells in different subjects. N = Normal; IH = Idiopathic haemochromatosis; IDA = Iron deficiency anaemia; $\beta T = \beta$-Thalassaemia; CA = Breast cancer, stages I, II, III and IV; B = Benign; INF = Benign inflammatory type; NIF = Benign non-inflammatory type; ------Normal upper limit.

**Figure 3** Distribution of the percentage of heart ferritin-positive T-cells in different subjects. N = Normal; B = Benign breast disease; CA = Breast cancer, stages I, II, III and IV; ------ Normal upper limit.

ferritin and 7.8% for heart ferritin-bearing T-cells was used, these being the 95% confidence limits for the normal group. Only 2 out of 20 normal subjects (10%) had higher levels of spleen ferritin-bearing T-cells. In contrast, 78% of cancer patients showed elevated values. Of these 39 patients, 13 of the 17 (76%) with stage I, all 11 with stage II, 11 of the 18 (63%) with stage III disease and all 4 patients with stage IV advanced disease had elevated values above the cut-off level (Figure 2). However, there was no relationship between the percentage positive cells and the clinical stage of malignancy. Although 7 out of 20 (35%) patients with benign breast disease did show increased levels, 6 of these were among the 7 patients with inflammatory breast disease, whereas only 1 out of 13 patients with non-inflammatory disease showed this phenomenon. None of the normal subjects had more than 7.8% heart ferritin-bearing T-cells, whereas 3 out of 8 patients with benign disease (38%) and 20 out of 30 cancer patients (67%) showed elevated values. There was no relationship between the percentage of heart ferritin-bearing T-cells and clinical stage.

There is no overall correlation between the percentage ferritin-positive T-cells and serum ferritin concentration. The presence of nodal involvement in breast cancer patients was not related to the percentage of ferritin-positive T-cells (Table I).

*Other T-cell surface antigens*

Measurement of Tac and transferrin receptors on T-cells in normal subjects and patients with breast disease showed no significant difference in their levels, whereas in cancer patients $13.4 \pm 6.0\%$ of T-cells were HLA-DR positive compared to $5.4 \pm 7.5\%$ in normal subjects ($P < 0.01$) and $11.5 \pm 5.0\%$ in patients with benign breast disease (NS) (data not shown). There was no significant difference in total T-cells, T-helper or B-cells between normal and breast disease patients. However, T-suppressor cells in cancer patients ($23.6 \pm 5.9\%$) were significantly increased ($P < 0.001$) compared to either normal subjects ($18.9 \pm 9.0\%$) or patients with benign breast disease ($17.5 \pm 7.3\%$) (data not shown).

### Discussion

In this study serum ferritin concentrations in normal subjects and patients with iron deficiency or overload are similar to those found previously (Jacobs *et al.*, 1972; Worwood, 1982). It also confirms other earlier reports (Marcus & Zinberg, 1975; Jacobs *et al.*, 1976) of high circulating ferritin levels in patients with breast cancer. There are many possible causes of raised serum ferritin levels in malignancy, including increased iron stores, release of ferritin from inflamed tissues and production of ferritin by proliferating tumour cells (Jacobs & Worwood, 1975). Although it is tempting to consider it as a tumour associated protein, the reason is still far from clear (Jacobs, 1984).

Measurement of T-lymphocyte surface ferritin shows that the mean percentage of ferritin-positive T-cells in breast cancer patients is significantly higher than in normal subjects and in patients with benign breast disease. Seventy to eighty percent of cancer patients have an increased percentage of ferritin-bearing T-cells, compared to less than 10% in the

**Table I** Distribution of the percentage of spleen ferritin-positive T-cells and node status in breast cancer patients

| *Node status* | *Number of patients* | *Ferritin bearing T-cells* $<7.04\%$ | $>7.04\%$ |
|---|---|---|---|
| – | 18 | 10 | 8 |
| + | 17 | 7 | 10 |

control group. These results are consistent with other investigations in patients with mammary carcinoma (Moroz et al., 1984; Bluestein et al., 1984) and other malignant diseases (Bluestein et al., 1984; Steinhoff et al., 1984; Papenhausen et al., 1984).

Of greater importance is the correlation between ferritin-bearing lymphocytes and the clinical stage of the disease; unfortunately there is some disagreement in this area. Moroz et al. (1984) found that the level of ferritin-positive cells in breast cancer patients was elevated in stages I, II and IV, but low in stage III. In our study, although there is a significant increase in spleen ferritin-bearing T-cells in all stages of breast cancer, this does not appear to be related to disease stage (Figure 2). However, there is an increased level in only 61% of stage III patients compared to stage I (76%), stage II (100%) and stage IV (100%). Moroz et al. (1984) hypothesised that this rather surprising finding in stage III patients may be due to deranged traffic of ferritin-bound lymphocytes, which accumulate at an extravascular site to be replaced in the blood by other lymphocytes, as suggested by the studies of De Sousa et al. (1978). Jacobs et al. (1984) found an elevated level of spleen ferritin-bearing lymphocytes in patients with Hodgkin's disease, but no relationship to disease stage. A similar result has also been reported in patients with head and neck cancer (Papenhausen et al., 1984).

Spleen ferritin-positive T-cells show no significant difference between the normal controls, patients with benign breast lesion, patients with iron deficiency anaemia and patients with idiopathic haemochromatosis. Although 8 out of 20 patients with benign breast disorders show elevated levels of T-cells bearing spleen ferritin, 6 of these are of an inflammatory type and the phenomenon may be a result of T-cell activation (Pattanapanyasat et al., 1987). This could also explain the increased number of HLA-DR positive cells in breast cancer patients.

This study shows that there are zero or low Tac-positive and transferrin receptor-positive T-cells in breast cancer patients. It is known that T-cell proliferation is dependent on the presence of Tac antigen (Uchiyama et al., 1981), transferrin receptor (Larrick & Cresswell, 1979), and HLA-DR antigen (Ko et al., 1979) and that resting T-cells possess few of these molecules. It is unusual to see an increase in the level of HLA-DR positive T-cells unrelated to other proliferative markers, but it may simply be that the degree of HLA-DR positivity found here is a response to mild stimulation and is certainly not comparable to that seen in mitogen stimulated T-cells (Ko et al., 1979; Pattanapanyasat et al. 1987) where levels are 5–6 times higher than in normal T-cells.

Similarly, heart ferritin-bearing T-cells show no relationship with the clinical disease stage. Measurements of T-cells bearing either spleen or heart ferritin do not appear to discrminate between the clinical stage of breast cancer. However, it is able to differentiate, to some extent, between patients with benign breast disease and patients with breast carcinoma, or between normal and cancer patients and it might help in identifying some patients with inflammatory or premalignant breast lesions.

It has been suggested that ferritin may play a crucial role in suppressing normal T-cell function (Matzner et al., 1979; Hancock et al., 1979). Our results show no correlation between T-cells bearing ferritin and serum ferritin concentration. This suggests that the increased number of T-cells bearing ferritin is not secondary to high circulating levels. Moreover, a low percentage of T-cells bearing ferritin has been demonstrated in both patients with homozygous beta-thalassaemia and hyperferritinaemia. Moroz et al. (1977b) found 4 patients with thalassaemia to be devoid of ferritin-bearing lymphocytes. Recently, Steinhoff et al. (1984) studied lymphocyte surface ferritin in patients known to have high levels of serum ferritin, such as haemochromatotic patients, patients with rheumatoid arthritis and patients with bacterial infection. None of them had elevated levels of lymphocyte-bearing ferritin.

At present, the precise subset of lymphocytes in breast cancer patients to which the ferritin-bearing lymphocytes belong is unknown. Cragg et al. (1984) showed that a minor fraction of both T-helper and T-suppressor cells have surface ferritin. In addition, our results show that the level of peripheral blood T-suppressor cells in patients with breast cancer is higher than that of normal subjects, though whether there is any connection with an increase in ferritin-bearing T-cells is not clear.

Although the functional significance of this phenomenon is unknown, it may prove to be another useful indicator of the presence of malignancy along with other tumour markers.

K.P. grategully acknowledges the financial support of the British Council.

### References

ALI, F.M.K., MAY, A., MCLAREN, G.D. & JACOBS, A. (1982). A two-step procedure for obtaining normal peripheral blood T-lymphocytes using continuous equilibrium density gradient centrifugation on Percoll. *J. Immunol. Methods*, **49**, 185.

BLUESTEIN, B.I., LUDERER, A.A., HESS, D. & 4 others (1984). Measurement of ferritin-bearing peripheral mononuclear blood cells in cancer patients by radioimmunoassay. *Cancer Res.*, **44**, 4131.

CONRADIE, J.D. & MBHELE, B.E. (1980). Quantitation of serum ferritin by enzyme-linked immunosorbent assay (ELISA). *South Afr. Med. J.*, **57**, 282.

CRAGG, S.J., HOY, T.G. & JACOBS, A. (1984). The expression of cell surface ferritin by peripheral blood lymphocytes and monocytes. *Br. J. Haematol.*, **57**, 679.

DE SOUSA, M., SMITHYMAN, A. & TAN, C. (1978). Suggested models of ecotaxopathy in lymphoreticular malignancy. *Am. J. Path.*, **90**, 497.

DORNER, M.H., SILVERSTONE, A., NISHIYA, K., DE SOSTOA, A., MUNN, C. & DE SOUSA, M. (1980). Ferritin synthesis by human T-lymphocytes. *Science*, **209**, 1019.

GUESDON, J.L., TERNYNCK, T. & AVRAMEAS, S. (1979). The use of avidin-biotin interaction in immunoenzymatic techniques. *J. Histochem. Cytochem.*, **27**, 1131.

HANCOCK, B.W., BRUCE, L., MAY, K. & RICHMOND, J. (1979). Ferritin, a sensitizing substance in the leucocyte migration inhibition test in patients with malignant lymphoma. *Br. J. Haematol.*, **43**, 223.

JACOBS, A. (1984). Serum ferritin and malignant tumours. *Med. Oncol. Tumor. Pharmacother.*, **1**, 149.

JACOBS, A., HODGETTS, J. & HOY, T.G. (1984). Functional aspects of isoferritins. In *Ferritins and Isoferritins as Biochemical Markers*, Albertini, A. *et al.* (eds) p. 113. Elsevier: Amsterdam.

JACOBS, A., JONES, B., RICKETTS, C., BULBROOK, R.D. & WANG, D.Y. (1976). Serum ferritin concentration in early breast cancer. *Br. J. Cancer*, **34**, 286.

JACOBS, A., MILLER, F., WORWOOD, M., BEAMISH, M.R. & WARDROP, C.A.J. (1972). Ferritin in the serum of normal subjects and patients with iron deficiency and iron overload. *Br. Med. J.*, **iv**, 206.

JACOBS, A. & WORWOOD, M. (1975). The biochemistry of ferritin and its clinical implications. In *Progress in Haematology*, **Vol. 9**, Brown, E.B. (ed). Grune and Stratton: New York.

JONES, B.M. & WORWOOD, M. (1978). An immunoradiometric assay for the acidic ferritin of human heart: Application to human tissues, cells and serum. *Clin. Chim. Acta*, **85**, 81.

KO, H.S., FU, S.M., WINCHESTER, R.J., YU, D.T.Y. & KUNKEL, H.G. (1979). Ia determinants on stimulated human T lymphocytes: Occurrence on mitogen- and antigen-activated T-cells. *J. Exp. Med.*, **150**, 246.

LARRICK, J.W. & CRESSWELL, P. (1979). Modulation of cell surface iron transferrin receptors by cellular density and state of activation. *J. Supramol. Struct.*, **11**, 579.

LEVY, R. & KAPLAN, H.S. (1974). Impaired lymphocyte function in untreated Hodgkin's disease. *New Engl. J. Med.*, **290**, 181.

MARCUS, D.M. & ZINBERG, N. (1975). Measurement of serum ferritin by radioimmunoassay: Results in normal individuals and patients with breast cancer. *J. Natl Cancer Inst.*, **55**, 791.

MATZNER, Y., HERSHKO, C., POLLIACK, A., KONIJN, A.M. & IZAK, G. (1979). Suppressive effect of ferritin on *in vitro* lymphocyte function. *Br. J. Haematol.*, **42**, 345.

MOROZ, C., GILER, S., KUPFER, B. & URCA, I. (1977*a*). Ferritin-bearing lymphocytes and T-cell levels in peripheral blood of patients with breast cancer. *Cancer Immunol. Immunother.*, **3**, 101.

MOROZ, C., LAHAT, N., BINIAMINOV, M. & RAMOT, B. (1977*b*). Ferritin on the surface of lymphocytes in Hodgkin's disease patients: A possible blocking substance removed by levamisole. *Clin. Exp. Immunol.*, **29**, 30.

MOROZ, C., KAN, M., CHAIMOF, C., MARCUS, H., KUPFER, B. & CUCKLE, H.S. (1984). Ferritin-bearing lymphocytes in the diagnosis of breast cancer. *Cancer*, **54**, 84.

PARRY, D.H., WORWOOD, M. & JACOBS, A. (1975). Serum ferritin in acute leukaemia at presentation and during remission. *Br. Med. J.*, **i**, 245.

PAPENHAUSEN, P.R., EMESON, E.E., CROFT, C.B. & BOROWIECKI, B. (1984). Ferritin-bearing lymphocytes in patients with cancer. *Cancer*, **53**, 267.

PATTANAPANYASAT, K., HOY, T.G. & JACOBS, A. (1987). The response of intracellular and surface ferritin following T-cell stimulation *in vitro*. *Clin. Science*, **73**, 605.

SARCIONE, E.J., SMALLEY, J.R., LEMA, M.J. & STUTZMAN, L. (1977). Increased ferritin synthesis and release by Hodgkin's disease peripheral blood lymphocytes. *Int. J. Cancer*, **20**, 339.

STEINHOFF, G., VAN DER HEUL, C., VAN EIJK, H., RICE, L. & ALFREY, C. (1984). Elevated lymphocyte surface ferritin in malignant diseases and mononucleosis infection. A lymphocyte ferritin antibody binding test. In *Ferritins and Isoferritins as Biochemical Markers*, Albertini, A. *et al.* (eds) p. 181. Elsevier: Amsterdam.

UCHIYAMA, T., BRODER, S. & WALDMAN, T.A. (1981). A monoclonal antibody (anti-Tac) reactive with activated and functionally mature human T-cell: 1. Production of anti-Tac monoclonal antibody and distribution of Tac(+) cells. *J. Immunol.*, **126**, 1393.

WORWOOD, M. (1980). Serum ferritin. In *Methods in Haematology, 1: Iron*, Cook, J.D. (ed) p. 59. Churchill Livingstone: London.

WORWOOD, M. (1982). Ferritin in human tissues and serum. *Clin. Haematol.*, **11**, 275.

WORWOOD, M., SUMMERS, M., MILLER, F., JACOBS, A. & WHITTAKER, J.A. (1974). Ferritin in blood cells from normal subjects and patients with leukaemia. *Br. J. Haematol.*, **28**, 27.